# Xanthurenic Acid in the Shell Purple Patterns of *Crassostrea gigas*: First Evidence of an Ommochrome Metabolite in a Mollusk Shell

**DOI:** 10.3390/molecules26237263

**Published:** 2021-11-30

**Authors:** Michel Bonnard, Bruno Boury, Isabelle Parrot

**Affiliations:** 1 IBMM, University of Montpellier, CNRS, ENSCM, 34095 Montpellier, France; michel.bonnard@umontpellier.fr; 2 TARBOURIECH-MEDITHAU, 34340 Marseillan, France; 3 ICGM, University of Montpellier, CNRS, ENSCM, 34095 Montpellier, France

**Keywords:** *Crassostrea gigas*, shell pigments, porphyrins, xanthurenic acid, ommochromes

## Abstract

Ommochromes are one of the least studied groups of natural pigments, frequently confused with melanin and, so far, exclusively found in invertebrates such as cephalopods and butterflies. In this study focused on the purple color of the shells of a mollusk, *Crassostrea gigas*, the first evidence of a metabolite of ommochromes, xanthurenic acid (XA), was obtained by liquid chromatography combined with mass spectrometry (UPLC-MS). In addition to XA and various porphyrins previously identified, a second group of high molecular weight acid-soluble pigments (HMASP) has been identified with physicochemical and structural characteristics similar to those of ommochromes. In addition, fragmentation of HMASP by tandem mass spectrometry (MS/MS) has revealed a substructure common to XA and ommochromes of the ommatin type. Furthermore, the presence of melanins was excluded by the absence of characteristic by-products among the oxidation residues of HMASP. Altogether, these results show that the purple color of the shells of *Crassostrea gigas* is a complex association of porphyrins and ommochromes of potentially ommatin or ommin type.

## 1. Introduction

Molluscan shell pigments are generally assigned to carotenoids, melanins and tetrapyrroles [1]. While the presence of carotenoids and few tetrapyrroles such as uroporphyrin and biliverdin are well established [1,2,3,4], the occurrence of melanins in shells of bivalves is apparently less common than generally expected, as illustrated by the recent work of S. Affenzeller et al. [5]. For instance, the black color of the adductor muscle scar of shells of the edible oyster *Crassostrea gigas,* initially hypothesized as a contribution of melanins by S. Hao et al. [6], was subsequently ruled out but without resolving the nature of this color. Recently, uroporphyrin and derivatives were identified in the mantle of *C. gigas* and the purple and dark patterns of its shell [7], constituting an evidence of the heme-based cellular respiration of *C. gigas* [8]. These represent only a small proportion of the overall acid-soluble pigments among which, the occurrence of ommochromes would corroborate the recent identification of genes associated with their biosynthetic pathways [9]. However, the precise chemical structure of ommochromes is generally unknown at present, their occurrence in a natural sample is usually postulated by the identification of specific biosynthetic metabolites such as 3-hydroxykynurenine (3-HK), 3-hydroxyanthranilic acid (3-HA) and XA [10,11,12,13].

Our previous study having established the presence of porphyrins [7], the present study focuses on the composition of porphyrins-free acid-soluble pigments of purple patterns of shells of *C. gigas* in order to establish the absence or presence of melanins and ommochromes, carotenoids were not considered, being the only group of acid-insoluble molluscan shell pigments [1,14,15,16]. Among know biosynthetic metabolites, only XA, a precursor and/or a degradation product of ommochromes [12], was identified from the resulting group of acid-soluble pigments. These also displayed physicochemical properties similar to those reported in the literature for ommochromes (insoluble in most aqueous and organic solvents without acidifier, absorption bands from 400 to 600 nm). In addition, they were constituted by a sub molecular unit common to that observed from the fragmentation of XA by tandem mass spectrometry. Finally, the absence of melanins was established by the method described by S. Affenzeller et al. [5]. Altogether, this leads us to consider the purple color of *C. gigas* as an association of acid-soluble porphyrins and a type of ommochromes.

## 2. Results

### 2.1. Identification of Xanthurenic Acid

**Figure 1:**
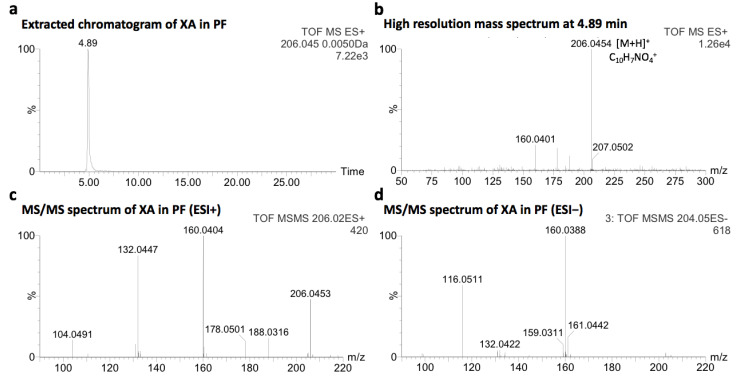

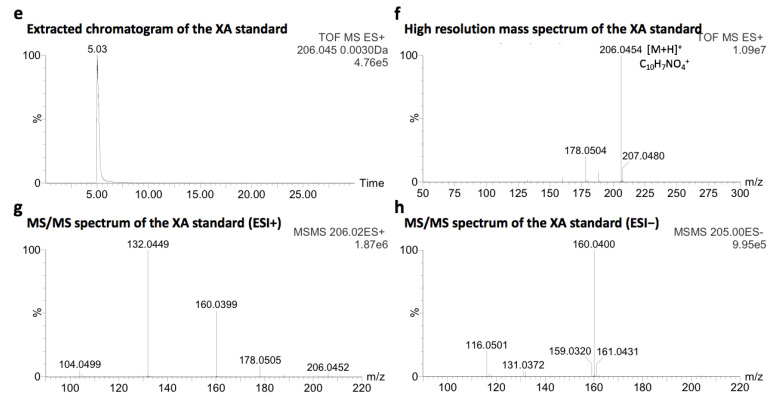
(**a**,**b**) Extracted chromatogram and high resolution mass spectrum of XA identified in PF, respectively; (**c**,**d**) MS/MS fragmentation spectra of XA identified in PF, in positive (ESI+) and negative (ESI−) ionization mode, respectively; (**e**,**f**) Extracted chromatogram and high resolution mass spectrum of the XA commercial standard, respectively; (**g**,**h**) MS/MS fragmentation spectra of the XA commercial standard, in positive (ESI+) and negative (ESI−) ionization mode, respectively.

After collection and decontamination, colorful purple fragments of shells of adult *C. gigas* were dissolved in aqueous hydrochloric acid (1M HCl_(aq)_) and filtered. A fraction of acid-soluble pigments, free of porphyrins, was obtained by preparative chromatography in opened system. The resulting fraction, named purple fraction (PF, 0.37 wt.%), was analyzed by UPLC-MS. The molecular formula of the compound eluted at 4.89 min, corresponding to C_10_H_7_NO_4_ ([M + H]^+^_obs_ at *m/z* 206.0454, Figure 1a,b), is consistent with XA ([M + H]^+^_calc_ at *m/z* 206.0453). The different retention times of XA in PF and the XA standard (4.89 and 5.03 min, respectively) is certainly due to the matrix effect. The identification of XA was further confirmed by the comparative UPLC-MS/MS analysis of a commercial standard (MS/MS spectra of XA in PF in Figure 1c,d, the pure XA standard in Figure 1e–h, mass spectra in ESI− and co-injected with the shell sample in Figure S1, adapted from [17]).

The identification of XA is of particular interest since it was described as a precursor as well as a side or degradation product of ommochrommes, exclusively related to their biosynthesis in invertebrates [12]. Consequently, the [M + H]^+^ signals of other known metabolite precursors and side products of possible acid-soluble molluscan pigments other than porphyrins (Figure S2), i.e., ommochromes and melanins, were searched from the UPLC-MS data of PF. Among these compounds, only two signals potentially corresponding to anthranilic acid and kynurenic acid were detected in PF at 3.71 and 4.50 min, respectively (Table S1), questioning the presence of melanins among the set of acid-soluble pigments in PF.

### 2.2. Characterization of the Purple Fraction of Acid-Soluble Pigments

The physicochemical properties of PF were investigated to define more precisely the chemical nature of its acid-soluble pigments. An important solubility was observed in alkali solution and in acidified solvents, especially in methanol containing HCl_(aq)_ (Table 1). Clearly, the solubility of PF is similar to that of ommochromes [16], being described in the literature as insoluble in almost all aqueous and organic solvents and slightly soluble in pure methanol but turning fully soluble when acidified with HCl [16,18].

**Figure 2:**
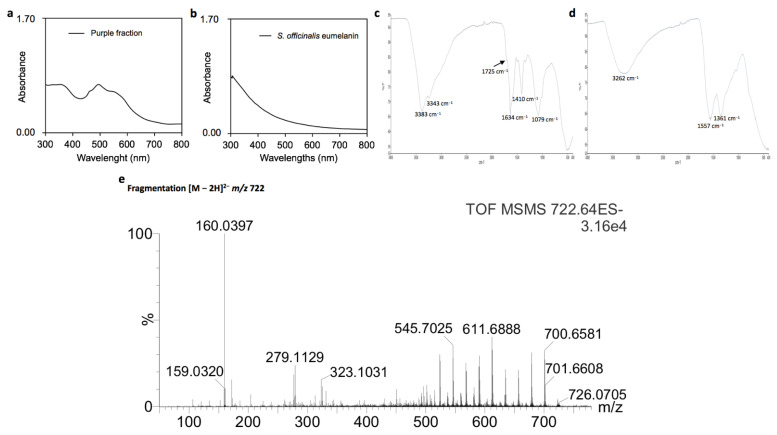
(**a**,**b**) Absorption spectra of PF and *Sepia officinalis* eumelanin, respectively; (**c**,**d**) FTIR spectra of PF and *Sepia officinalis* eumelanin, respectively; (**e**) MS/MS spectrum of the major acid-soluble pigment from PF ([M − 2H]^2−^ at *m/z* 722), adapted from reference [17].

The absorption spectrum of PF in the UV-visible region (Figure 2a) was characterized by an absorption band at 360 nm and a large band from 400 to 600 nm, with λ_max_ at 464, 496 and 552 nm. This absorption profile is comparable to those of ommochromes (a large band from 400 to 600 nm and a smaller around 310 or 380 nm depending on the pH and chemical structure) [16,18,19,20], but strongly differs from those synthetic and natural melanins of different sources, all characterized by a continuous decreasing absorption towards the visible region without specific absorption band from 400 to 800 nm [21] (Figure 2b).

The infrared (IR) spectrum of PF (Figure 2c) is also comparable to those of ommochromes either ommatin or ommin types [20,22,23,24]. Among characteristic bands, the broad band at 3000–3500 cm^−1^ may be representative of carboxylic acid function. The bands at 1634 cm^−1^ potentially correspond to C-O stretching vibration of carboxylic acid function, and 1410 cm^−1^ to N-H bending vibrations. In addition, the band at 1725 cm^−1^ is in agreement with that described as specific to ommochromes of the ommatin type [20,24]. In contrast, the IR spectrum of *Sepia officinalis* eumelanin is characterized by broad bands at 3262 cm^−1^, 1557 cm^−1^ and 1361 cm^−1^ (Figure 2d) as already mentioned in the literature [25]. Given the structural diversity of melanins, additional bands can be observed, some of which are common to ommochromes due to the presence of carboxylic acid, amide, amine, aromatic amine and phenolic functions [25].

The analysis of PF by UPLC-MS in negative ionization mode (more sensitive for acidic compounds compared to the positive ionization mode), was characterized by ions with a state of charge of 2 in the *m/z* 700−770 range. The major ion at *m/z* 722 investigated by MS/MS was characterized by multiple neutral losses of CO_2_ (≥9), being representative of carboxylic acid groups (Figure 2e). In addition, the intense product ion at *m/z* 160, corresponding to C_9_H_6_NO_2_^−^, was also observed in the fragmentation spectrum of XA (Figure 1d), suggesting a common sub-structural unit. These results were systematically observed for other ions with a state of charge of 2 in the *m/z* 700–770 range (Figure S3).

### 2.3. Comparative Analysis with Natural Melanin

**Figure 3:**
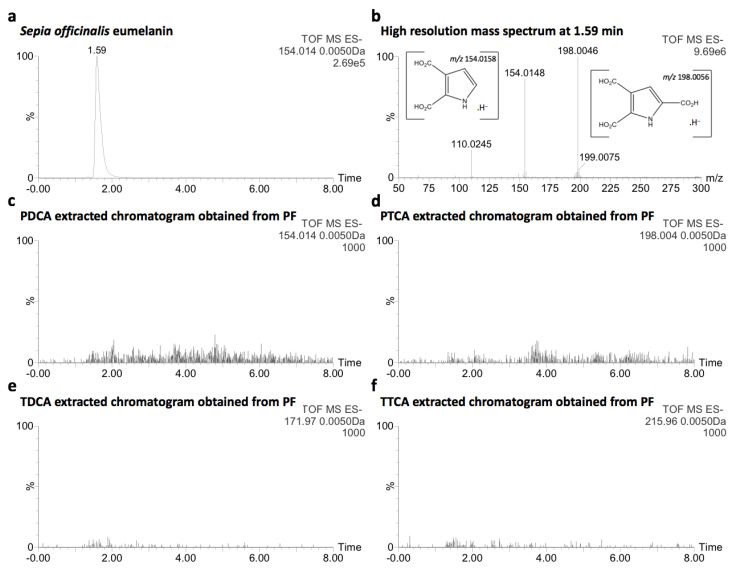
(**a**,**b**) Extracted chromatogram of *Sepia officinalis* eumelanin oxidation products and the corresponding high resolution mass spectrum; (**c**–**f**) Extracted chromatograms of melanins oxidation products from PF, background noise.

The discrimination of melanins from ommochromes in a natural sample is challenging and often lead to confusion from the macroscopic (granular morphology) to the molecular point of view (polycarboxylic and polyaromatic structure) [19,26,27,28]. In order to establish whether or not melanins are present in PF, an oxidation method [29], recently applied by S. Affenzeller et al. [5], was used since it has allowed to investigate the presence of melanins in the black adductor muscle scar of shells of *C. gigas*. Briefly, after alkaline oxidation of PF with a 30% H_2_O_2_ aqueous solution (pH > 10), the resulting products were analyzed by UPLC-MS in negative ionization mode and compared to eumelanin of *Sepia officinalis* treated in the same conditions. From the latter, the molecular ions of pyrrole-2,3-dicarboxylic acid (PDCA) [M − H]^−^ at *m/z* 154.0148 and pyrrole-2,3,5-tricarboxylic acid (PTCA) [M − H]^−^ at *m/z* 198.0046 were detected at 1.59 min (Figure 3a,b). In contrast, none of these oxidation products of melanins, including those of pheomelanin (thiazole-4,5-dicarboxylic acid and thiazole-2,4,5-tricarboxylic acid), were observed in PF (Figure 3c–f), supporting the absence of melanins in PF among the set of acid-soluble pigments. The two other well-known melanin markers (4-amino-3-hydroxyphenylalanine and 3-amino-tyrosine [25], [M − H]^−^_calc_ at *m/z* 195.0770) were not detected in both samples (Figure S4), possibly due to sample preparation by solid phase extraction.

## 3. Discussion

In mollusks, like in other living organism, “*similar shell colors can arise from different pigments*” [3]. Conversely, a given group of pigments can produce different shell colors especially those with a complex polymeric structure varying according to the living organism and its environment. In this study, we noted that at least two groups of acid-soluble pigments were involved in the purple color of shells of the oyster *C. gigas*. Among possible pigments supported by the genes associated with their biosynthesis (carotenoids, melanins, ommochromes and porphyrins) [9], acid-soluble porphyrins (uroporphyrin I or III and derivatives) were recently established [7,8]. Besides, after separation of porphyrins from PF, the absence of animal melanins and corresponding known metabolites is established here, in line with the recent study of S. Affenzeller et al. [5]. If animal melanins are deposited in the shell purple patterns of *C. gigas*, they are not among acid-soluble pigments.

**Figure 4:**
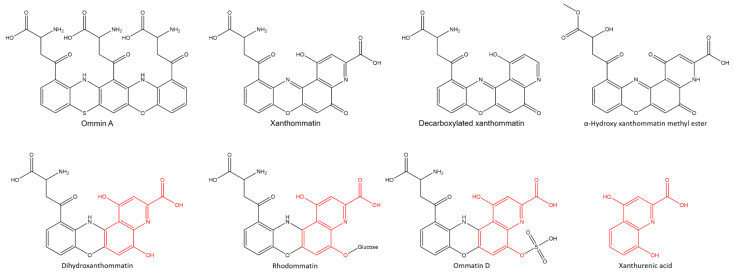
Structure of ommin A, some known ommatins and xanthurenic acid.

Pioneering studies on ommochromes have proposed a subdivided classification according to their dialysis profile: ommatins (rather dialyzable), ommins (almost non-dialyzable) and ommidins (intermediate) [18]. To date, the structures of approximately fourteen natural ommatins have been established but the structure of ommins and ommindins is poorly described. For example, the well accepted structure of ommin A [18,30] (Figure 4) is solely based on chemical properties and elemental determination [19,31]. Besides, ommidins have completely disappeared from experimental investigations subsequent to the work of B. Linzen in 1974 [19]. Only a recent review points out their possible occurrence in invertebrates [18]. In the ommochrome literature, the identification of XA is a decisive parameter. For examples, the red, red-brown and yellow pigments of wings of *Junonia coenia* (common buckeye) were attributed to dihydroxanthommatin, ommatin D and xanthommatin (ommochromes of the ommatin type) but none were detected by MS/MS liquid chromatography, in contrast to XA [10,32]. Indeed, in invertebrates, XA is described as a key metabolite of the biosynthesis of ommochromes [12,13,18,19], exclusively related to this biological route [12]. To date, two main biosynthetic pathways of ommochrome pigments have been proposed, both involving XA. The first involves the condensation of XA with 3-hydroxyanthranilic acid and/or 3-hydroxykynurenine [11]. The second involves only 3-hydroxykynurenine (3-HK) as an intermediate by condensation of two units [12]. In this case, XA is described as a side product of the intramolecular cyclisation of 3-HK or as a degradation product of higher ommochromes of the ommatin type. Since XA was identified among known ommochrome metabolite precursors and intermediates, such a similar process could occur in the case of shell purple patterns of *C. gigas*. Starting from tryptophan as the initial precursor, the genes responsible for XA biosynthesis in the ommochrome pathway, i.e., tryptophan-2,3-dioxygenase, kynurenine formamidase and kynurenine-3-monooxygenase [18], have been identified in the genome of *C. gigas* [33]. Only 3-HK transaminase was not identified yet (3-HK → XA). Therefore, in the case of this study XA may either be a metabolite produced in excess or a degradation product of multiple possible origin (during dissolution in aqueous acid conditions with light exposition, biosynthesis or during the development of the shell). From a structural point of view, a XA sub molecular unit can be distinguished from the phenoxazine unit of ommatins (red in Figure 4), a characteristic also observed from the acid-soluble pigments of PF investigated by tandem mass spectrometry, where none of known ommochromes were identified (Figure S5). The molecular weight of the acid-soluble pigments of PF, higher than those of ommatins, and the numerous carboxylic acid groups of their structure may be consistent with the polymeric/oligomeric nature of ommins described in the literature. However, there is no available commercial standard to confirm this assumption. Since XA was identified in PF, it may originate from a degradation of acid-soluble pigments of PF. Experiments on the mantle edge epithelium by a non- or soft-destructive process could also give reliable information on the structure of acid-soluble pigments of PF, and could allow the correlation of a potential function in living conditions. Experiments using matrix assisted laser desorption ionization combined with mass spectrometry conducted on solid state samples (PF or shell purple fragments) could give reliable information on species not detected by MS liquid chromatography as was the case for allomelanin from black oat [34]. To date, the strong absorption of acid-soluble pigments of PF in the visible region suggests a potential protection against light but other properties could emerge, eventually related to an oxidation process as observed in the production of uroporphyrin and derivatives [7,8].

Whatever is the definitive structure of these compounds; the high number of carboxylic groups is reminiscent to those of uroporphyrin and derivatives. Related to the mineralization process of the shell, their occurrence is consistent with the binding of pigments to the calcite part of the shell via an ionic carboxylate-Ca^2+^ bond, that is also suitable for the transport and fixation of calcium in the shell. It remains to be elucidated whether this was selected by nature, in order to ensure the binding of pigments designed for a specific function, or whether pigments are a carboxylic-rich by-product of the physiology of the animal resulting in their coincidental accumulation on the shell surface. A potential perspective of this study would lie in the selective extraction of XA from purple shells of *C. gigas* as a natural source substituting synthetic XA, and its use to study neurological and tryptophan metabolism disorders [35,36,37].

## 4. Materials and Methods

### 4.1. Shell Fragments

Approximately 1 kg of shell fragments were collected by hand on living adult oysters in August 2017 (Thau lagoon, Marseillan, France, GPS coordinates: 43.382127, 3.555193). Shell fragments were rinsed with tap water at the farm and transported to the laboratory. Shell fragments were extensively rinsed with tap water and suspended in 0.0155M NaOCl_(aq)_ with sonification and regular manual stirring (1:10 wt/v, 120 min). Shell fragments were rinsed several times and suspended in demineralized water with sonification and regular manual stirring (1:10 wt/v, 120 min). Shell fragments were rinsed several times with demineralized water and dried in oven (overnight, 40 °C). Shell fragments were sorted in three classes according to their color. Only fully purple shell fragments were used in this study. Samples were stored in the dark at 25 °C before use.

### 4.2. Identification of XA in PF

Approximately 10 g of decontaminated purple fragments of shells of *C. gigas* were dissolved in 1M HCl_(aq)_ under magnetic stirring (1:20 wt/v, 30 min, 700 RPM). The solution of acid-soluble pigments was obtained after filtration on a glass sintered filter (POR 4) filled with Fontainebleau sand. The solution of acid-soluble pigments (40 mL) was deposited on a C_18_ grafted silica gel (approximately 40 g) previously equilibrated with 1M HCl_(aq)._ After deposition, decalcification was performed with 80 mL 1M HCl_(aq)_ followed by 80 mL 0.1% TFA. Separative elution was monitored by fluorescence at λ_ex_ 400 nm and conducted with 420 mL of ultrapure water/acetonitrile (80:20 *v*/*v* + 0.1% TFA). The resulting non-photoluminescent PF was freeze-dried and weighted (0.37 wt.%). Separation was followed with 140 mL acetonitrile +0.1% TFA. The resulting photoluminescent fraction (porphyrins) was freeze-dried and weighted (<0.1 wt.%). The purple fraction (1 mg) was solubilized in 200 μL of 1M HCl_(aq)_, filtered on a polyethersulfone syringe filter (0.22 μm) and analyzed on a UPLC Synapt G2-S system (Waters Corporation, Milford, MA, USA) equipped with an electrospray ionization source (UPLC-DAD-Q-ToF-HRMS, Waters Corporation, Milford, MA, USA). UV-vis spectra were recorded with a UPLC LG 500 nm DAD detector from 200 to 500 nm with a resolution of 1.2 nm and a sampling rate of 20 points/sec. Separation was carried out using a 150 × 2.1 mm Kinetex 2.6 μm EVO C18 100 Å reverse stationary phase, operating at 30 °C with a constant flow rate of 0.5 mL/min using ultrapure water (0.055 μS/cm) and acetonitrile HPLC grade as eluents both containing 0.1% formic acid. Mass spectra were recorded in the *m/z* range of 50 to 3000 with a ZQ spectrometer fitted with Micromass Q-Tof spectrometer operating at capillary voltage of 3 kV and cone voltage of 30 V, using phosphoric acid as an internal standard. Masslynx software (version V4.1, Waters Corporation, Milford, MA, USA) was used for instrument control and data processing. Samples were kept at 10 °C in the autosampler. Appropriate blank analysis was performed before each sample (V_inj_: 10 μL). Blank TIC chromatogram was systematically subtracted to the corresponding sample TIC chromatogram before data processing. Separation was performed with a gradient system: 0 to 50% acetonitrile in 20 min, followed by 50 to 100% acetonitrile in 5 min, followed by 100% acetonitrile in 1 min, followed by 100% ultrapure water in 0.1 min and finally 4.9 min with 100% ultrapure water. A solution of 10 mg/mL of XA was prepared in 1 mL of 1M HCl_(aq)_ under magnetic stirring (60 min, 700 RPM), followed by filtration on polyethersulfone syringe filter (0.22 μm), XA being slightly soluble in water. The resulting solution was analyzed by UPLC-DAD-Q-ToF-HRMS according to the method previously employed. MS/MS experiments were performed in collision-induced dissociation mode with a trap collision energy ramp from 15 to 40 eV and using auto transfer collision energy of 2 eV. Argon was used as the collision gas.

### 4.3. Characterization of PF

The qualitative estimation of the PF solubility was conducted at 1 mg/mL (60 min, 500 RPM, 20 °C), followed by centrifugation (20 min, 4400 RPM). Absorption spectra were recorded from 200 to 800 nm using UV-1800 spectrophotometer, 10 mm optical path length (Shimadzu Corporation, Kyoto, Japan). Appropriate auto zero on solvent blank was performed before each measurement. The absorption spectrum of PF was obtained with 1 mg in 1mL of 1M HCl_(aq)_ and diluted by a factor 10 and 100. The absorption spectrum of *S. officinalis* was obtained with 1 mg in 1mL of 1M HCl_(aq)_ and filtered on polyethersulfone syringe filter 0.22 μm (very slightly soluble). IR spectra were recorded using a spectrum two FTIR spectrometer (ATR mode, PerkinElmer, Waltham, MA, USA). UPLC-MS/MS was conducted according to the previously described method. Automatic MS/MS experiments were conducted using auto transfer collision energy of 2 eV. Argon was used as the collision gas. Collision-induced was performed in dissociation mode. MS/MS range was set from 50 Da to 1500 Da. The number of fragmented compounds was set at 3 × 4. MS/MS fragmentation was set to switch after 2 s with a scan time of 0.1 s. Peak detection was used in intensity based peak detection mode and peak detection window with a charge state tolerance of *m/z* 0.2. The trap MS/MS collision energy was set according to a ramp from 30 to 50 eV. The cone voltage was set at 40 V. The collision energy was set according to a ramp from low mass (50 Da, 10–20 eV) to high mass (1500 Da, 80–140 eV).

### 4.4. Comparative Analysis with Natural Eumelanin

The oxidation was conducted with 10 mg of PF, ultrapure water (1 mL), 1M K_2_CO_3(aq)_ (3.75 mL) and 30% H_2_O_2(aq)_ (250 μL), under magnetic stirring (20 h, 500 RPM, 20 °C). A volume of 500 μL of 10% Na_2_SO_3(aq)_ was added. A volume of 550 μL of the solution was mixed with 140 μL of 6M HCl_(aq)_. After centrifugation (20 min, 4,400 RPM), the supernatant was collected and purified by solid phase extraction (Strata-X 200 mg, Phenomenex Inc., Torrance, CA, USA)). Conditioning was conducted with methanol (6 mL) followed by ultrapure water (6 mL). After sample loading, washing was conducted with 0.3% aqueous formic acid (3 mL). Elution was conducted with methanol (3 mL) and ethyl acetate (3 mL). The collected fraction was evaporated under a constant flux of argon for approximately 5h. After evaporation, the solid residue was solubilized in ultrapure water (200 μL) and analyzed by UPLC-Q-ToF-HRMS with the previously described Water alliances UPLC Synapt G2-S system in electrospray negative ionization mode (Waters Corporation, Milford, MA, USA). Separation was carried out using a 100 × 2.1 mm Kinetex 1.7 μm EVO C18 100 Å reverse stationary phase, operating at 45 °C with a constant flow rate of 0.2 mL/min using ultrapure water (0.055 μS/cm) and acetonitrile HPLC grade as eluents both containing 0.1% formic acid. Mass spectra were recorded in the *m/z* range of 50 to 1500 with a ZQ spectrometer fitted with Micromass Q-Tof spectrometer (Waters Corporation, Milford, MA, USA) operating at capillary voltage of 2.4 kV and cone voltage of 30 V, using phosphoric acid as an internal standard. Masslynx software (version V4.1, Waters Corporation, Milford, MA, USA) was used for instrument control and data processing. Samples were kept at 10 °C in the autosampler (V_inj_: 10 μL). Separation was performed with a gradient system: 0 to 20% acetonitrile in 20 min, followed by 20 to 100% acetonitrile in 1 min, followed by 100% acetonitrile in 2 min, followed by 100% ultrapure water in 0.1 min and finally 4.9 min with 100% ultrapure water. The entire process was repeated with 10 mg of *Sepia officinalis* eumelanin.

### 4.5. Chemicals

Acetonitrile and methanol HPLC grade (≥99.9%) were purchased from Fisher scientific (Merelbeke, Belgium). Trifluoroacetic acid HPLC grade was purchased from Fisher scientific (Loughborough, UK). C_18_ grafted silica for flash high throughput purification was purchased from Supelco (Bellefonte, PA, USA, batch SP98226). Fontainebleau sand, hydrochloric acid 37%, hydrogen peroxide 30% and sodium sulfite were purchased from VWR Chemicals (Leuven, Belgium). Formic acid ULC/MS grade (99%) was purchased from Biosolve (Valkenswaard, The Netherlands). Sodium hypochlorite 9.6% was purchased from Notilia (Nîmes, France). *Sepia officinalis* eumelanin was purchased from Sigma-Aldrich (Burlington, MA, USA, batch #103H1023). Ultrapure water (0.055 μS/cm) was obtained by pre-filtration and reverse osmosis system (Labostar Pro TWF, Evoqua water technologies, Pittsburgh, PA, USA). Xanthurenic acid was purchased from Interchim (Montluçon, France, batch V0226P002).

## Figures and Tables

**Table 1:** Qualitative estimation of PF solubility ^1^, data from [17].

Solvents	Solubility
Acetic acid (1M, aqueous)	Fully soluble
Acetone	Not soluble
Cyclohexane	Not soluble
Chloroform	Not soluble
Dichloromethane	Not soluble
Diethyl ether	Not soluble
Ethanol	Slightly soluble
Ethyl acetate	Not soluble
Isopropyl alcohol	Not soluble
Hexane	Not soluble
HCl (1M, aqueous)	Fully soluble
Methanol	Slightly soluble
Methanol containing 1% of 12M HCl_(aq)_	Fully soluble
Methanol containing 0.1% of 12M HCl_(aq)_	Fully soluble
Sodium hydroxide (1M, aqueous)	Fully soluble
Ultrapure water	Slightly soluble

^1^ Tested at 1 mg/mL, for 60 min at 500 RPM and 20 °C.

## Data Availability

Not applicable.

## Supplementary Materials

The following are available online, Figure S1: Identification of XA in PF, Figure S2: Known metabolite precursors and side products of ommochromes and melanins searched in PF, Figure S3: UPLC-MS/MS analysis of PF, Figure S4: Absence of melanin oxidation products in both *S. officinalis* eumelanin and PF samples, Figure S5: Absence of ommin A and some known ommatins in PF; Table S1: Identification of ommochrome metabolites in PF.

## Sample Availability

Samples of the compounds are available from the authors.

## Abbreviations

3-HA	3-hydroxyanthranilic acid
3-HK	3-hydroxykynurenine
ESI−	electrospray negative ionization mode
ESI+	electrospray positive ionization mode
FTIR	Fourier transform infrared spectroscopy
HMASP	high molecular weight acid-soluble pigments
IR	infrared
MS/MS	tandem mass spectrometry
PDCA	pyrrole-2,3-dicarboxylic acid
PF	purple fraction
PTCA	pyrrole-2,3,5-tricarboxylic acid
TDCA	thiazole-4,5-dicarboxylic acid
TFA	trifluoroacetic acid
TTCA	thiazole-2,4,5-tricarboxylic acid
UPLC-MS	liquid chromatography mass spectrometry
UPLC-MS/MS	liquid chromatography tandem mass spectrometry
XA	xanthurenic acid
