# Supplementary material for: Xanthurenic Acid in the Shell Purple Patterns of Crassostrea gigas: First Evidence of an Ommochrome Metabolite in a Mollusk Shell
Source: Molecules. 2021 Nov 30;26(23):7263. doi: 10.3390/molecules26237263 (PMC8658808; doi:10.3390/molecules26237263)
Supplement: Supplementary file 1 [file molecules-26-07263-s001.zip › molecules-1446714-supplementary.pdf]

molecules

MDPI

Supplementary Materials

Xanthurenic Acid in the Shell Purple Patterns of *Crassostrea gigas*; First Evidence of an Ommochrome Metabolite in a Mollusk Shell

Michel Bonnard [1,2], Bruno Boury [3,*] and Isabelle Parrot [1,*]

[1] IBMM, University of Montpellier, CNRS, ENSCM, Montpellier, France; michel.bonnard@umontpellier.fr (M.B.); isabelle.parrot-smietana@umontpellier.fr
[2] TARBOURIECH-MEDITHAU, Marseillan, France
[3] ICGM, University of Montpellier, CNRS, ENSCM, Montpellier, France
* Correspondence: bruno.boury@umontpellier.fr (B.B.); isabelle.parrot-smietana@umontpellier.fr (I.P.)

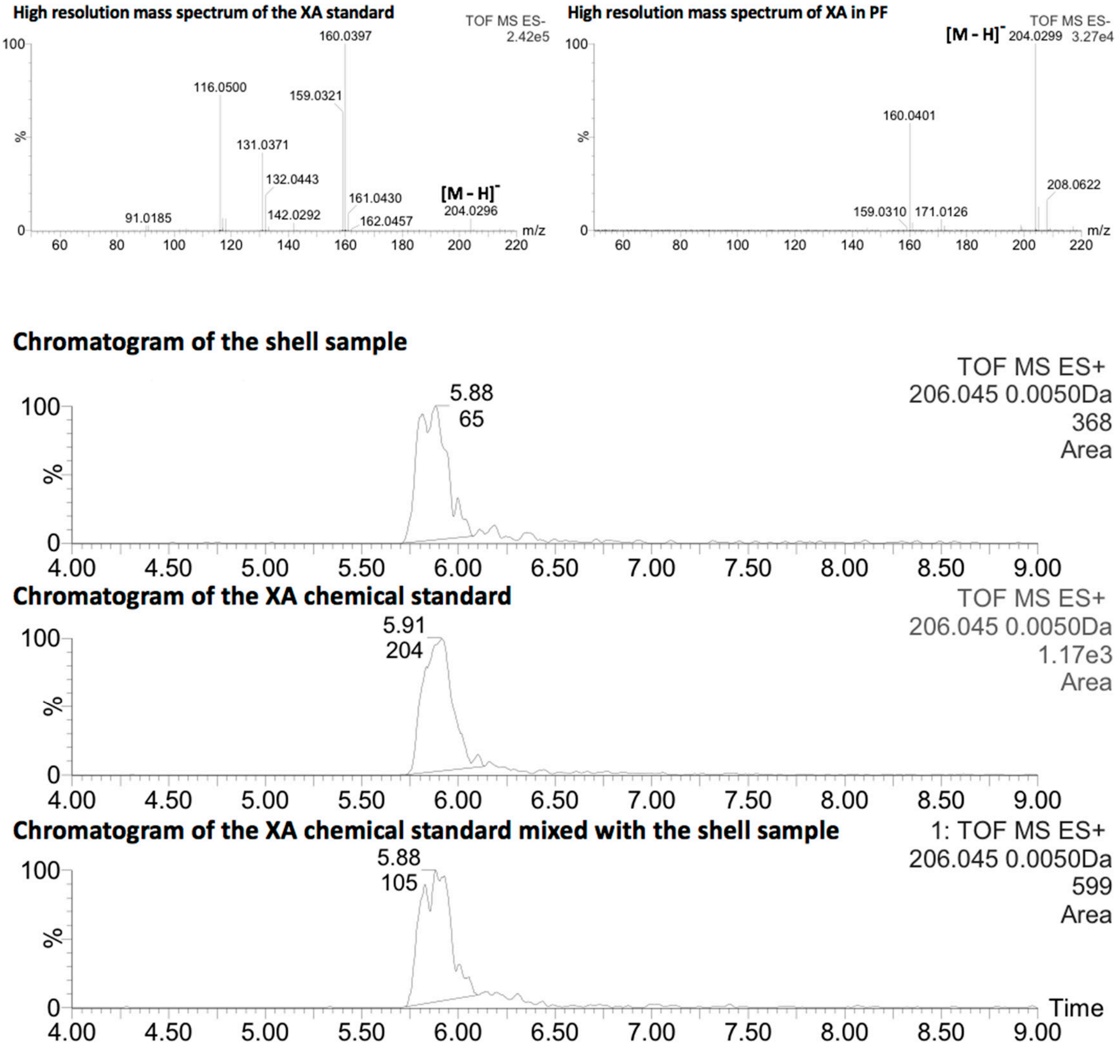

Figure S1. Identification of XA in PF. High resolution mass spectra of the XA standard and XA in PF obtained in ESI- and co-injection of the XA standard with the shell sample (extracted chromatogram of XA from: the shell sample, Peak area 65, from the chemical standard, Peak area 204, from a 50:50 V/V mixture of the shell sample and the chemical standard diluted by a factor 865, Peak area 105).

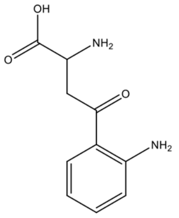

Kynurenine
$C_{10}H_{12}N_2O_3$
$[M + H]^+_{calc}$ *m/z* 209.0926
Not detected

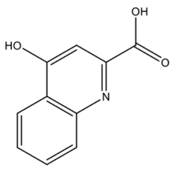

Kynurenic acid
$C_{10}H_7NO_3$
$[M + H]^+_{calc}$ *m/z* 190.0504
Detected

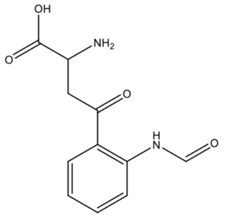

Formylkynurenine
$C_{11}H_{12}N_2O_4$
$[M + H]^+_{calc}$ *m/z* 237.0875
Not detected

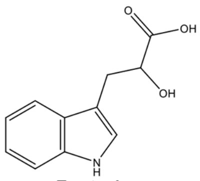

Tryptophan
$C_{11}H_{12}N_2O_2$
$[M + H]^+_{calc}$ *m/z* 205.0977
Not detected

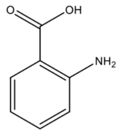

Anthranilic acid
$C_7H_7NO_2$
$[M + H]^+_{calc}$ *m/z* 138.0555
Detected

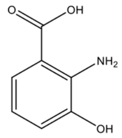

3-hydroxyanthranilic acid
$C_7H_7NO_3$
$[M + H]^+_{calc}$ *m/z* 154.0504
Not Detected

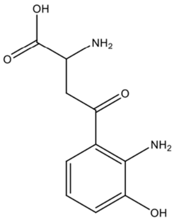

3-hydroxykynurenine
$C_{10}H_{12}N_2O_4$
$[M + H]^+_{calc}$ *m/z* 225.0875
Not detected

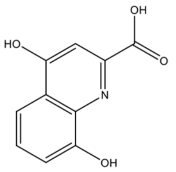

Xanthurenic acid
$C_{10}H_7NO_4$
$[M + H]^+_{calc}$ *m/z* 206.0453
Detected

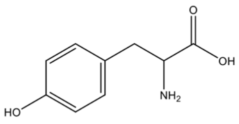

_L_-Tyrosine (eumelanin)
$C_9H_{11}NO_3$
$[M + H]^+_{calc}$ *m/z* 182.0817
Not detected

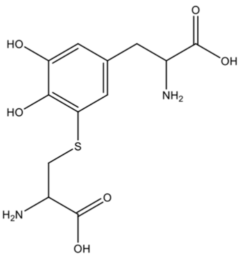

5-cys-dopa (pheomelanin)
$C_{12}H_{16}N_2O_6S$
$[M + H]^+_{calc}$ *m/z* 317.0807
Not detected

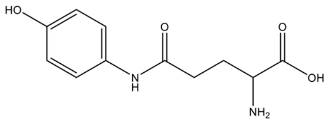

4-glutaminyhydroxybenzene (mushroom)
$C_{11}H_{14}N_2O_4$
$[M + H]^+_{calc}$ *m/z* 239.1032
Not detected

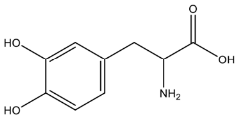

_L_-Dopa (eumelanin)
$C_9H_{11}NO_4$
$[M + H]^+_{calc}$ *m/z* 198.0766
Not detected

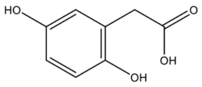

Homogentisic acid (pyomelanin)
$C_8H_8O_4$
$[M + H]^+_{calc}$ *m/z* 169.0501
Not detected

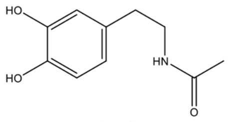

N-acetyl-dopamine (insect-melanin)
$C_{10}H_{13}NO_3$
$[M + H]^+_{calc}$ *m/z* 196.0974
Not detected

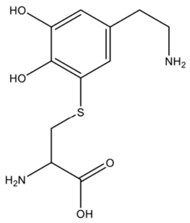

5-_S_-cys-dopa (neuromelanin)
$C_{11}H_{16}N_2O_4S$
$[M + H]^+_{calc}$ *m/z* 273.0909
Not detected

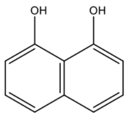

1,8-dihydroxynaphtalene (fungi)
$C_{10}H_8O_2$
$[M + H]^+_{calc}$ *m/z* 161.0602
Not detected

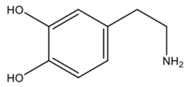

Dopamine (neuromelanin)
$C_8H_{11}NO_2$
$[M + H]^+_{calc}$ *m/z* 154.0868
Not detected

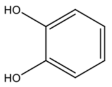

Catechol (plant)
$C_6H_6O_2$
$[M + H]^+_{calc}$ *m/z* 111.0446
Not detected

Figure S2. Known metabolite precursors and side products of ommochromes and melanins searched in PF.

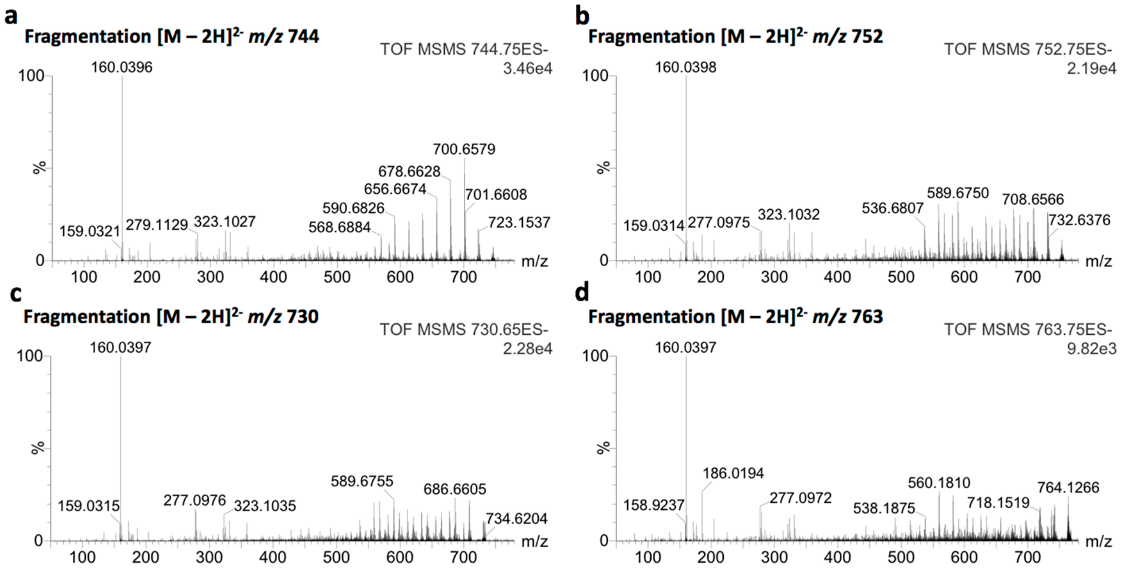

Figure S3. UPLC-MS/MS analysis of PF. (**a**–**d**) Fragmentation spectra of acid-soluble pigments of PF (negative ionization mode).

Table S1. Identification of ommochrome metabolites in PF.

Compounds	Molecular formula	Retention time (min)	*m/z* [M + H]$^+_{obs}$	*m/z* [M + H]$^+_{calc}$	Mass accuracy (ppm)
Anthranilic acid	$C_7H_7NO_2$	3.71	138.0548	138.0555	Δ −5.1
Kynurenic acid	$C_{10}H_7NO_3$	4.50	190.0505	190.0504	Δ 0.5 ppm

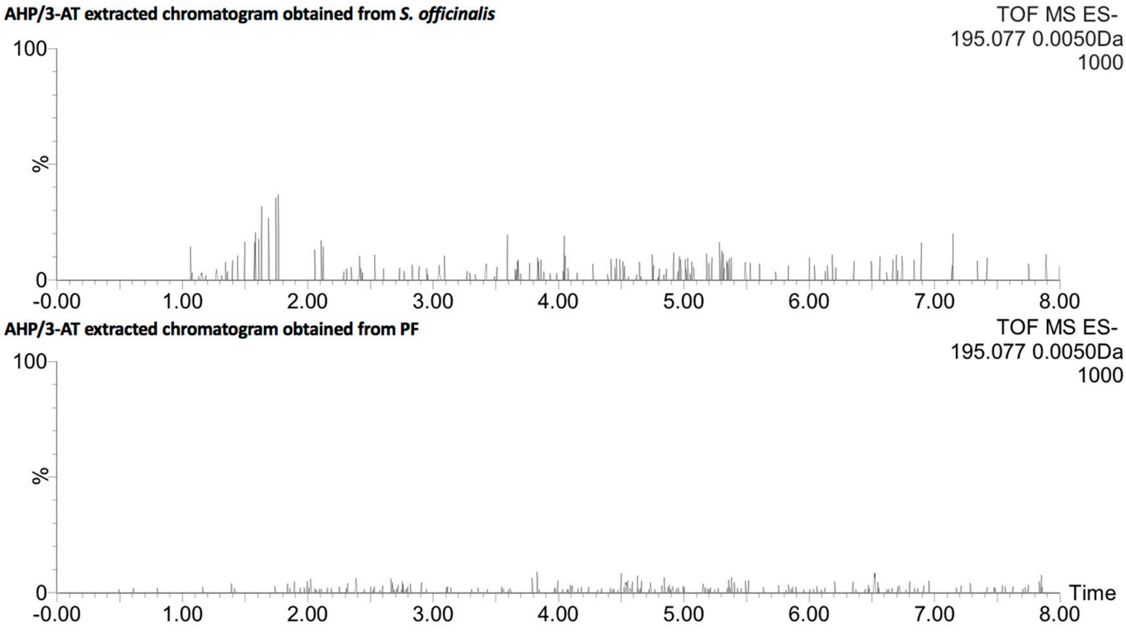

Figure S4. Absence of melanin oxidation products in both *S. officinalis* eumelanin and PF samples.

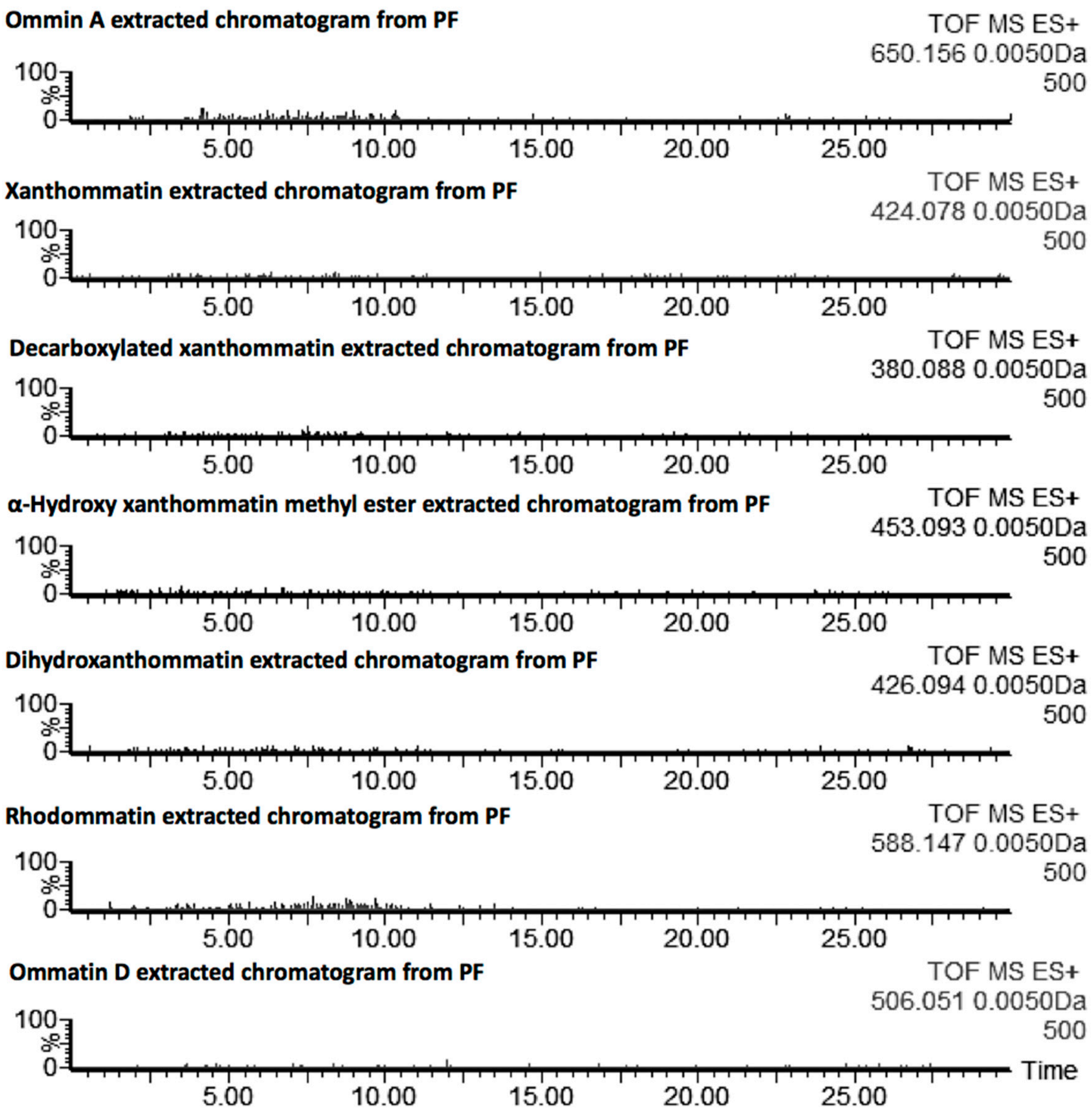

Figure S5. Absence of ommin A and some known ommatins in PF.

Copyright: © 2021 by the authors. Licensee MDPI, Basel, Switzerland. This article is an open access article distributed under the terms and conditions of the Creative Commons Attribution (CC BY) license (http://creativecommons.org/licenses/by/4.0/).
